# Pilot Safety and Feasibility Study of Non-invasive Limb Proprioceptive Cerebellar Stimulation for Epilepsy

**DOI:** 10.3389/fneur.2021.675947

**Published:** 2021-08-17

**Authors:** Ronald M. Harper, Dieter Hertling, Ashley Curtis, Eberhardt K. Sauerland, Christopher M. De Giorgio

**Affiliations:** ^1^Department of Neurobiology, David Geffen School of Medicine, Univeersity of California, Los Angeles, Los Angeles, CA, United States; ^2^Department of Neurology, Olive View Medical Center, University of California, Los Angeles, Los Angeles, CA, United States; ^3^Department of Neurology, David Geffen School of Medicine, University of California, Los Angeles, Los Angeles, CA, United States; ^4^Department of Physiology, University of Nevada, Reno, NV, United States

**Keywords:** neuromodulation, SUDEP, proprioception, seizures, vibration, epilepsy, neurostimulation, cerebellum

## Abstract

Cerebellar stimulation reduces seizures in animals and in humans with drug-resistant epilepsy. In a pilot safety and feasibility study, we applied continuous cutaneous vibratory stimulation (limb proprioceptive cerebellar stimulation) to foot limb proprioceptive receptors to activate cerebellar, pontine, and thalamic structures in drug-resistant epilepsy patients for 8-h nocturnally up to 6-months after a 4-week pre-treatment control baseline. Seizure frequency was evaluated during the baseline control period, and at 6, 12, and 24 weeks after the control recordings. Five-subjects completed at least the first 6-week treatment. At 12-weeks, the median reduction in seizure frequency was −27.8% (mean reduction = −22.3%). Two subjects continued for 24 weeks, with a decline of −44.1 and −45.4%. This pilot study provides support for further clinical studies into the safety and efficacy of limb proprioceptive cerebellar stimulation for epilepsy.

## Introduction

There is evidence that peripheral sensory stimuli suppress epileptic discharges and seizures in animals and humans (1–6). Such peripheral stimulation activates proprioceptive and sensory pathways which project to cerebellar and thalamic structures. Direct electrical, optogenetic, and transcranial magnetic stimulation of the cerebellum has been explored as a means to reduce seizures since the pioneering studies of Cooper and others in the 1970's (7–13). More generalized non-invasive procedures, including hypothermia, transcranial and direct current stimulation of other brain areas, and electrical stimulation of cutaneous surfaces served by trigeminal and vagal nerves have been introduced as well (14–19). Cerebellar regulatory processes are central to protection and recovery from prolonged apnea and extreme hypotension (20, 21), conditions central to Sudden Unexpected Death in Epilepsy (SUDEP) (22, 23). The frequency of generalized tonic-clonic seizures is also a principal risk factor in SUDEP susceptibility (24). Devices which protect against peri-ictal apnea, hypotension and seizures may theoretically reduce SUDEP risk and reduce seizures in people with drug resistant epilepsy.

New means to reduce the number of seizures and protect against peri-ictal apnea are needed to reduce SUDEP risk in those with drug-resistant epilepsy. Recruitment of cerebellar structures to suppress ictal events may provide such a reduction though peripheral stimulation of proprioceptive receptors *via* vibration applied externally to peripheral limbs. Such an intervention offers a well-tolerated means to also reduce the severity of apnea, hypopnea and bradycardia accompanying ictal events in a fashion similar to that demonstrated in apnea of prematurity, using limb proprioceptive stimulation (25). Animal models also show that stimulation of the sole of the foot reduces seizures in a kainic acid model of epilepsy (26). Given the evidence for protection against apnea in humans, and the antiepileptic effect in animal models, we initiated a trial of non-invasive limb proprioceptive cerebellar stimulation in subjects with drug resistant epilepsy.

## Materials and Methods

Participants were recruited from the neurology clinic at Olive View-UCLA Medical Center, a public teaching hospital in the Greater Los Angeles area. Inclusion criteria included ages 18-55 and at least three seizures per month. After institutional review board approval and written informed consent by the patient or guardian, five subjects, aged 22-36 years-old (4 males, 1 female) with drug resistant epilepsy were enrolled in this pilot safety and feasibility study of non-invasive limb proprioceptive cerebellar stimulation. Table 1 shows demographic and clinical data for the study group. No changes in antiepileptic drugs were allowed unless needed for seizure safety. All subjects had failed at least three antiseizure medications.

**Table 1:** Summary of Subject Data.

**Subject**	**Baseline seizure frequency**	**Duration of epilepsy**	**Seizure types**	**Etiology**	**SUDEP-7R score**
	**Seizures per month (month = 30 days)**	**Age of onset**			**Range 0-10**
A	63.3	23 years	Multiple seizure types	Tuberous sclerosis Lennox-Gastaut	7
		Onset at birth	Bilateral tonic clonic Focal, impaired awareness		
B	620.1	21 yearsOnset at age 6 months	Multiple seizure types Bilateral tonic clonic,	Severe epileptic encephalopathy Lennox-Gastaut 6p25.1 mutation	7
C	49.7	21 years	Multiple seizure types	Lennox-Gastaut	7
		Onset at age 7 months	Bilateral tonic clonic, Focal, impaired awareness		
D	3.7	5 yearsOnset at age 26 years	Focal, impaired awareness	Unknown etiology	4
E	99.3	31 years	Multiple seizure types	Cortical dysplasia	8
		Onset at age 5 years	Bilateral tonic clonic Focal, impaired awareness		

*Identifiers removed to protect personal health information and confidentiality*.

**Figure 1:**
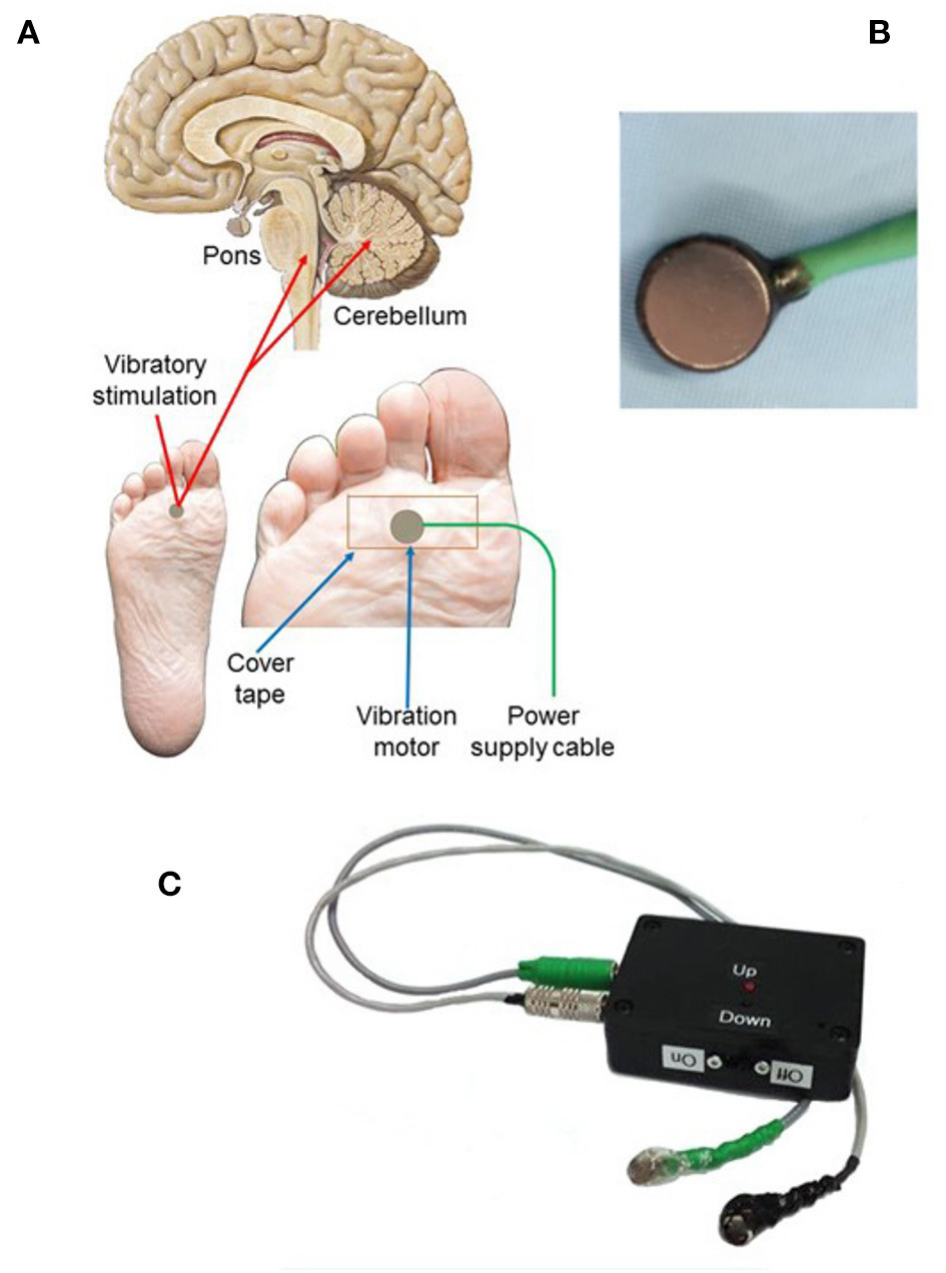
Vibratory placement, brain targets, and stimulation components of limb proprioceptive device. **(A)** Placement of vibrator motor on the sole of the foot, using non-allergenic tape for adherence; power supply leads provide power to the vibratory motor at 3.0 V. Central targets for vibration include the cerebellum and dorsal pons. **(B)** Vibrator motor with power leads. **(C)** Power supply containing two 1.5VAA batteries with two levels of vibratory stimulation (up = 3.0 V; down = 1.5 V; vibration was always at 3.0 V in this study). Component sources and vibratory motor specifications are outlined in Supplementary Material.

After a 4-week pretreatment baseline, continuous limb proprioceptive cerebellar stimulation was initiated. Stimulation consisted of cutaneous 128 Hz vibration through 12 mm disc motors delivered to the sole of the foot for 8-h nightly (Figure 1 and Supplementary Material). Patients or their caregivers tracked seizures using a seizure calendar. Scores from Patient-Reported Outcomes Measurement Information System (PROMIS) indicating sleep integrity and daytime sleepiness were administered at the end of the 24-week period (27).

At enrollment, subjects underwent a SUDEP-7 inventory to identify risk factors for SUDEP (28, 29). The SUDEP-7 score is a weighted score of seven risk factors associated with SUDEP, based on findings associated with risk for SUDEP identified in a prospective cohort study (30). Seizure frequency was initially counted as seizures per day, then converted to seizures per month, defined as 30 days per month.

## Results

Table 1 summarizes data for the five subjects. Identifiers that could lead to subject identification, such as actual assigned subject number are not included to ensure confidentiality. All subjects were at high risk for SUDEP, with a mean SUDEP-7 score of 6.8/9 (range 5.0-10.0, normal = 0) (28, 29). During the control 4-week baseline period, the mean seizure frequency was 167.1 seizures per month (range = 3.7-620.1/month).

**Figure 2:**
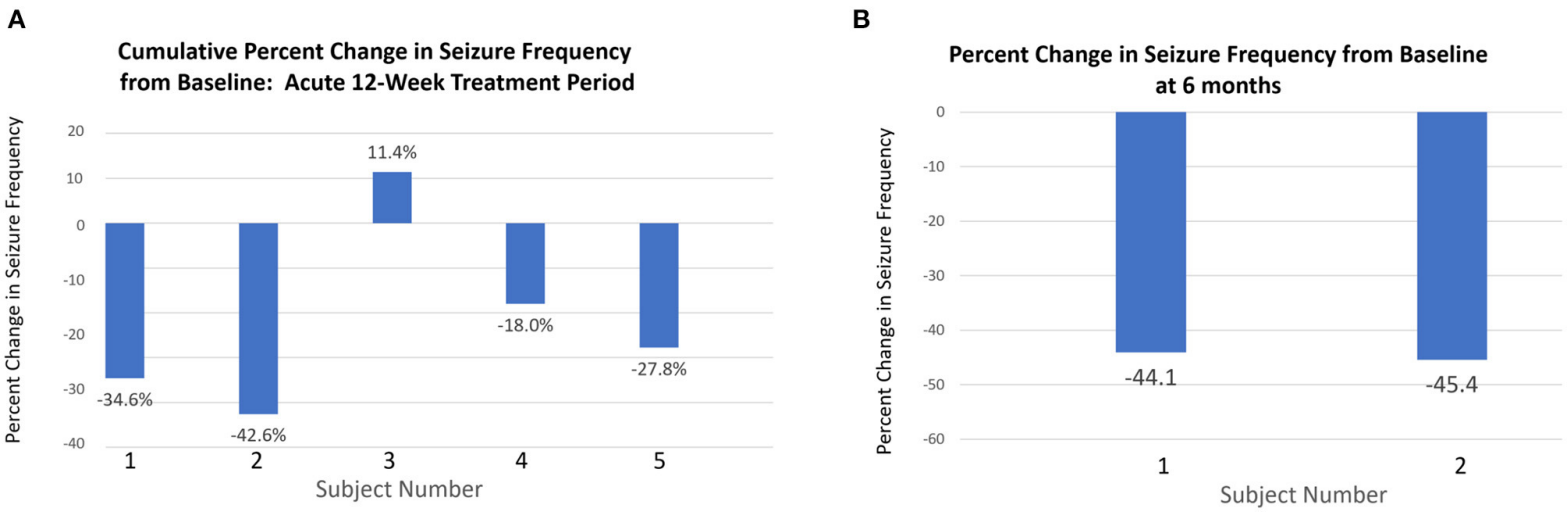
Changes in seizure frequency at 12 weeks and 6 months. **(A)** Percentage change in total seizure frequency at 12 weeks relative to baseline for all patients. **(B)** Two subjects (1 and 2) who continued with the intervention for 6 months showed greater percentage reduction in seizure frequency from baseline to 6 months than at 12 weeks.

Following 6-weeks post-baseline nocturnal vibration, median change in seizure frequency was −27.8% (mean = 23.8%, range = +3.7 to −42.7%). After 12-weeks, the cumulative median change in seizure frequency was −27.8%, (mean = −22.3%, range = −17.1 to −42.7%). Two subjects continued using the device for 6-months. After 6-months of limb proprioceptive stimulation, the percent change in seizure frequency for these two subjects was −44.1 and −45.4% (Figure 2). Both subjects had severe epileptic encephalopathy with intellectual disability and frequent generalized tonic clonic seizures.

The device was well-tolerated. No skin irritation was reported. No serious device-related or adverse events were reported. PROMIS scores at 6-months following onset of intervention trials showed a reduction in daytime sleepiness.

## Discussion

In this pilot safety and feasibility study, non-invasive limb proprioceptive cerebellar stimulation was safe and well-tolerated. Stimulation was associated with a −27.8% median reduction (−22.3% mean reduction) in seizure frequency after 3-months of stimulation. Two subjects continued to 6-months, and both experienced >40% reduction in seizure counts. This outcome emerged despite a high rate of seizures in the study group. The outcomes compare favorably with transcutaneous vagal nerve electrical stimulation [for vagal stimulation: −23.4% after 28 days; −34.2% after 20 weeks; (19)], and offer benefits of mild vibration rather than electrical stimulation.

Electrical stimulation of the cerebellum or excitation of the cerebellar fastigial nucleus reduce seizures significantly (8). A role for the cerebellum in triggering or suppressing seizures has repeatedly been suggested [for review, see (31)]. Proprioceptive stimulation, with ascending neural signaling *via* posterior column pathways, activates deep cerebellar nuclei, including the fastigial nuclei critical for such influences over seizures. Functional magnetic resonance imaging studies indicate that proprioceptive stimulation triggered by limb movements activates cerebellar deep nuclei and parabrachial pontine structures, as well as thalamic and insular regions, all significant areas for regulating seizure expression (32).

The proprioceptive stimulation may exert ancillary benefits for patients with epilepsy in addition to reducing seizure frequency. Apnea, hypoxemia, and bradycardia play key roles in the terminal mechanisms of SUDEP (22, 23). Interventions to reduce seizures and apnea, and provide cardiovascular support are urgently needed to reduce SUDEP risk. Cerebellar and brainstem structures that mediate recovery from apnea and hypotension (20, 21) demonstrate substantial injury and volume loss in people with epilepsy who later succumb to SUDEP (33). Non-invasive proprioceptive stimulation using a device similar to that described here reduces the severity of apneas, hypopneas, and bradycardic episodes in infants with apnea of prematurity and periodic breathing, presumably operating through cerebellar contributions to respiratory motor and autonomic coordination (25, 34). Since breathing and cardiovascular issues can add to neural injury accompanying seizure discharge, benefits in seizure reduction may be supplemented by respiratory and cardiac support.

## Limitations

The primary purpose of this study was to evaluate safety and tolerability, with a secondary aim of preliminary efficacy. The principal limitation was the small number of subjects, resulting from logistic constraints of prolonged data acquisition in a pilot study. The constraints also did not allow more prolonged, or different patterns of vibratory exposure, e.g., daytime exposure, which may have resulted in greater effectiveness. The small number and open nature of the investigation precludes definitive conclusions on efficacy, or which type of epilepsy would be most responsive to stimulation.

This report provides initial data that non-invasive limb proprioceptive cerebellar stimulation is a safe and novel intervention that may reduce seizure frequency in people with drug resistant epilepsy. The device is minimal risk, with low possibility for discomfort or skin injury, or interference with pacemakers, and is well-tolerated. Non-invasive limb proprioceptive cerebellar stimulation has the potential to reduce seizure frequency in people with epilepsy, and has been shown to reduce the risk of apnea, hypopnea, and associated bradycardia in premature infants (25) and spinal cord-injured patients (35). The vibratory intervention is minimally invasive, does not injure the skin as is possible with sustained electrical stimulation, and is very well-tolerated by sleeping individuals.

## Conclusions

Non-invasive limb proprioceptive cerebellar stimulation was safe and well-tolerated. Preliminary data from this small cohort with severe epilepsy at risk for SUDEP indicate subjects experienced a median change in cumulative seizure frequency of −27.8% (mean = −22.3%) at 12-weeks, with reductions of as much as 45.4% with more prolonged stimulation. The intervention offers a non-invasive and low-cost means to lower the number of seizures with minimal disruption to sleep. Further investigation of limb non-invasive cerebellar stimulation to reduce seizures in drug resistant epilepsy is indicated.

## Data Availability Statement

The raw data supporting the conclusions of this article will be made available by the authors, without undue reservation.

## Ethics Statement

The studies involving human participants were reviewed and approved by Olive View Medical Center IRB. The patients/participants provided their written informed consent to participate in this study.

## Author Contributions

RH and CD participated in conceptualization, design, analysis, and writing. ES was involved in conceptualization and writing. DH created electrode patches and assisted in spreadsheet creation, data entry, and analysis. AC was involved in patient recruitment, data entry, and analysis. All authors contributed to the article and approved the submitted version.

## Conflict of Interest

The University of California has filed for a provisional patent for the device described in the manuscript, listing RH, CD, and ES as inventors. The remaining authors declare that the research was conducted in the absence of any commercial or financial relationships that could be construed as a potential conflict of interest.

## Publisher's Note

All claims expressed in this article are solely those of the authors and do not necessarily represent those of their affiliated organizations, or those of the publisher, the editors and the reviewers. Any product that may be evaluated in this article, or claim that may be made by its manufacturer, is not guaranteed or endorsed by the publisher.
