# Supplementary material for: Pilot Safety and Feasibility Study of Non-invasive Limb Proprioceptive Cerebellar Stimulation for Epilepsy
Source: Front Neurol. 2021 Aug 17;12:675947. doi: 10.3389/fneur.2021.675947 (PMC8415900; doi:10.3389/fneur.2021.675947)
Supplement: Supplementary file 1 [file Data_Sheet_1.docx]

**Supplementary Material- vibratory motor details, construction, and implementation**

**Vibratory motor details**

**The source of the vibratory motors is as follows:**

KOTL **Jinlong Machinery & Electronics**

**JINLONG MACHINERY (HK) LIMITED**

**11/F, Tai Yau Building**

**181 Johnston Road**

**Wanchai, Hong Kong**

**Tel: +852 3002-4470**

**Email: sales@jinlong-machinery.com**

**USA SALES OFFICE:**

**Jinlong Machinery & Electronics, Inc.**

**535 Dean Street**

**Suite 804**

**Brooklyn, New York**

**11217 USA**

**Email: sales01@jinlong-machinery.com**

Model Number:C1234B016F

Description

<http://www.vibration-motor.com/products/pancake-vibrator-motors/coin-motor-C1234B016F.html>

| **Summary Of Specifications Vibratory Motor** | |
| --- | --- |
| Technology Type: | BRUSH |
| Diameter (mm): | 12.0 |
| Thickness (mm): | 3.4 |
| Rated Voltage (Vdc): | 3.0 |
| Operating Voltage (Vdc): | 2.7~3.3 |
| Rated Current MAX (mA): | 80 |
| Typical Current (mA): | 48 |
| Rise Time (ms) MAX *: | 90 |
| Fall Time (ms) MAX *: | 50 |
| Rated Speed (rpm): | 9000 |
| Vibration Force (G): | 2.00 |

**Power Supply**

Custom-made In-House:

Two AA 1.5 volt batteries, arranged in a serial configuration with a tap to switch between 1.5 volts or 3.0 volts (1.5 volt levels were used in an earlier infant study) provided an approximate 128 Hz vibration of the motor. The 3.0-volt configuration provides a higher g force and was used exclusively in this study. Outputs were fused to prevent the possibility of excessive current.

**Construction of device**

The vibratory motor leads are soldered to flexible multi-stranded cable which, in turn, are soldered to ¼” pin connectors for the power supply. The vibratory motor and lead configuration are covered by epoxy to solidify the connections.

**Implementation**

The flat surface of the vibratory motor is applied to the sole of the foot, as indicated in Figure 1 of the manuscript, and covered with allergen-free tape. Vibration is continuous, at approximately 128 Hz when 3.0-volt current is supplied. The vibratory motor was applied at bedtime, and continuously for 8 hr; the device is removed on morning wakening.
